# Ergonomics in Interventional Radiology: Awareness Is Mandatory

**DOI:** 10.3390/medicina57050500

**Published:** 2021-05-14

**Authors:** Francois H. Cornelis, Leo Razakamanantsoa, Mohamed Ben Ammar, Raphael Lehrer, Idriss Haffaf, Sanaa El-Mouhadi, Francois Gardavaud, Milan Najdawi, Matthias Barral

**Affiliations:** 1 Department of Interventional Radiology and Oncology, Tenon Hospital, Sorbonne Université, 75020 Paris, France; leo.razakamanantsoa@aphp.fr (L.R.); mohamed.benammar@aphp.fr (M.B.A.); lehrer.raphael@gmail.com (R.L.); ydriss92@hotmail.fr (I.H.); francois.gardavaud@aphp.fr (F.G.); milan.najdawi@aphp.fr (M.N.); matthias.barral@aphp.fr (M.B.); 2 Department of Radiology, Saint Antoine Hospital, Sorbonne Université, 75012 Paris, France; sanaa.el-mouhadi@aphp.fr

**Keywords:** computed tomography, ergonomics, interventional radiology, musculoskeletal disorders, radiation exposure

## Abstract

Ergonomics in interventional radiology has not been thoroughly evaluated. Like any operators, interventional radiologists are exposed to the risk of work-related musculoskeletal disorders. The use of lead shielding to radiation exposure and the lack of ergonomic principles developed so far contribute to these disorders, which may potentially affect their livelihoods, quality of life, and productivity. The objectives of this review were to describe the different situations encountered in interventional radiology and to compile the strategies both available to date and in development to improve ergonomics.

## 1. Introduction

Interventional radiology is now considered an effective option for treating a wide range of conditions [1,2]. It provides pain relief, improvement in function or quality of life, and prolonged survival [1,3,4,5]. Additionally, interventional radiology comprises a complex work environment with intensive psychological and physiological challenges [6]. As patient safety is a constant priority for interventional radiologists (IRs), most of them neglect basic ergonomic principles during procedures as well as their own health, as already observed in surgery [7,8,9].

To further optimize the performance and well-being of IRs, and meet the needs and demands of the patients and care, research in ergonomics is now mandatory in interventional radiology [10]. Ergonomics studies human interactions with elements. This has led to the search for a reduction in work-related injuries, performance errors, and loss of productivity [11]. IRs, like any physician who wears protective garments and stands during procedures, are exposed to musculoskeletal disorders (MSDs), which may ultimately lead to work-related stress syndrome [8,11]. Despite increased awareness of the importance of ergonomics, it is currently insufficiently incorporated into everyday IR practices. The objectives of this narrative review were to describe the different situations encountered in interventional radiology when performing procedures under various image guidance and to compile the strategies available to date to improve ergonomics in daily practice and in the future.

## 2. Materials and Methods

A literature search was performed on MEDLINE/PubMed focused on articles published between January 2000 and March 2021. The terms ‘‘interventional radiology’’ OR ‘‘surgery’’ AND ‘‘ergonomics’’ were used. The search was limited to the English language and covered all studies available until the search date. Study titles and abstracts were screened by the primary author, with studies of potential relevance progressing to full text screening. Where there was uncertainty regarding a study’s eligibility for inclusion, two additional authors were consulted and, through discussion, a decision was made on whether to include the study. A total of 84 articles were identified. Finally, 25 original articles were selected based on their relevance. Additionally, all cited references were analyzed to include additional papers related to the search and previously excluded by the initial review. A total of 62 articles were included in this review article, which follows the Scale for the Assessment of Narrative Review Articles [12]. All data were extracted by the primary author and peer-reviewed by two others. Key findings of the studies were extracted and narrated using a descriptive narrative synthesis method to facilitate the integration of results derived from a variety of methodologies.

## 3. Work-Related Musculoskeletal Disorders

### 3.1. Definition

Injuries occur when the load placed on a tissue exceeds the capacity of that tissue or when repeated exposure to lower loads, or risk factors, interferes with the ability of the body to recover [13,14,15,16]. Accumulation of trauma to the muscles and tendons result in chronic conditions identified as MSDs. Several regions, including the neck, upper back, lower back, shoulder, and wrist/hand, may be affected [8,16,17,18].

### 3.2. Risk Factors

**Figure 1:**
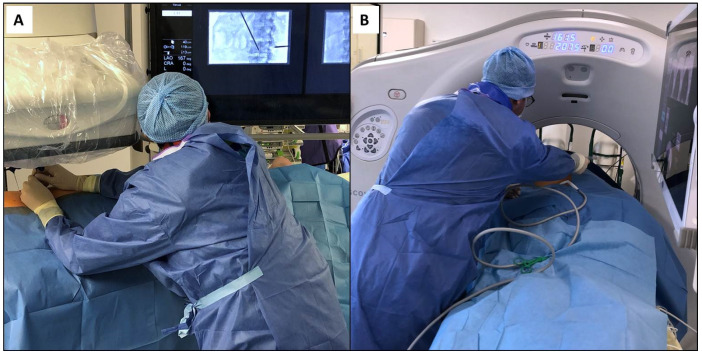
Percutaneous procedures performed under image guidance often require grip strength with instruments, bending for adequate needle positioning, and cervical rotation to visualize the screens: (**A**) vertebroplasty performed under cone-beam computed tomography; (**B**) cryoablation performed under computed tomography guidance. The resulting body contortion potentially leads to discomfort and musculoskeletal disorders.

In interventional radiology, the use of anti-X aprons, in association with awkward postures and non-ergonomic working conditions, might cause the onset of MSDs [19]. The prevalence of neck and back pain at least once a week ranges from 50% to 60% for those who use lead aprons frequently [8,20]. In a study involving interventional cardiologists, almost 30% developed upper extremity injuries [21]. Orme et al. identified women as presenting an increased risk of musculoskeletal pain [22], more specifically reported in the wrist or hand [17,23]. Age and cumulative exposure to stress at work are risks of developing MSDs [24]. However, up to 80% of younger physicians may also develop MSDs [24]. This may be due to their limited experience and operational skills, resulting in higher grip strength with instruments or excessive body contortion (Figure 1) or ergonomic settings adjusted to the senior’s specification [24]. Interestingly, residents or young surgeons complain significantly more of MSDs compared with seniors [9]. However, a bias toward under-reporting discomfort may exist due to the general culture or the survivor effect, whereby only healthier surgeons continue to operate [25,26].

### 3.3. Consequences

Work-related MSDs can cause pain and physical discomfort during procedures as well as time off work [13,14,15,16]. Unfortunately, work-related MSDs can also lead to work-related stress syndrome, known as burnout [11,27,28,29]. Burnout was defined by Davila et al. [30] as “a unique affective multidimensional response to stress, the core components of which are emotional exhaustion, physical fatigue, and cognitive weariness”. Burnout has adverse physical and psychological effects that may be correlated with musculoskeletal pain, changes in pain perception, fatigue, cardiovascular health, and depression [15,30,31]. In a recent study performed on 569 surgeons, those who felt physical discomfort reported significantly lower satisfaction with their work (*p* = 0.024), higher burnout (*p* = 0.005), and significantly higher callousness toward people (*p* < 0.001) than those not fearing loss of career longevity [15]. One additional consequence may be that the youngest might be deterred from interventional radiology, especially when they are aware of the risk of MSDs, as was observed in surgery [27]. There is thus a need to further explore risk situations in interventional radiology; evaluate their effects on productivity, patient care, and patient satisfaction; and identify interventions that can promote the wellness of IRs.

## 4. Situations Encountered in Interventional Radiology and Existing Solutions

### 4.1. Ultrasound-Guided Procedures

The awkward postures of the trunk, neck, and upper extremities used to perform ultrasound-guided procedures, as well as sustained or forceful gripping and downward force applied with the transducer, contribute to symptoms of discomfort and risk of injury [32]. An association with the number of individual studies per month (>100 scans), average scan time > 25 min, posture, high-pressure handgrip, and short stature (height < 63 inches) was observed with MSDs in the hand and wrist [33,34]. In addition, posture, axial twisting, and excessive reach during exams can lead to back, neck, and shoulder pain [35]. A recent study demonstrated that work-related MSDs in sonographers is highly prevalent, with 86% of those surveyed being affected [34]. Sonographers’ pain is more severe and worsens at a greater rate compared with others. IRs should vary their exam postures throughout their working day. Hand stretching exercises can help reduce muscle tension [36]. Alternating hand-holding the transducer can also reduce cumulative strain on the dominant hand. Arm abduction should be limited and neutral body positioning maintained, aided by positioning the monitor directly in front of the operator. The use of indirect lighting can limit visual fatigue.

### 4.2. Procedures Performed under Angiography and Cone-Beam Computed Tomography

**Figure 2:**
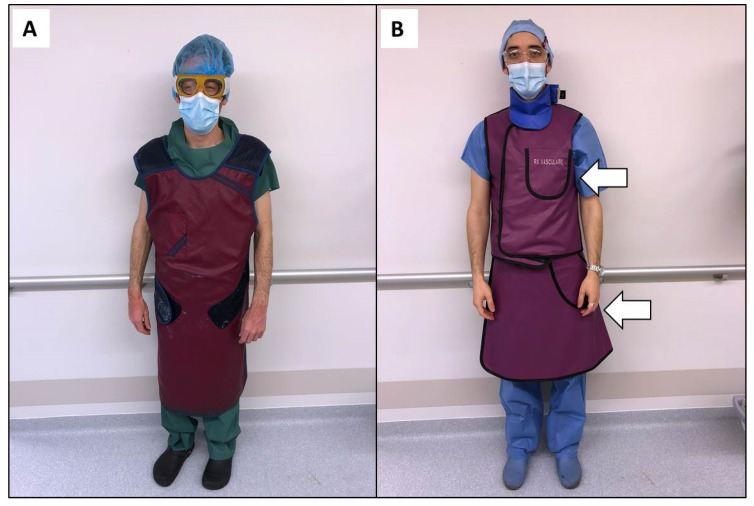
The lead apron is a heavy piece of radiation protection that should be worn by all staff working in the angio suite but it increases the risk of musculoskeletal disorders (**A**). A lighter two-part coat (arrows) helps to distribute the weight across the shoulders and waist and must fit properly to improve radiation protection (**B**).

In the angio suite, IRs must comply with recommendations to limit the radiation exposure to themselves [37,38]. A central piece of shielding is the lead apron. The lead apron is a heavy piece (up to 15 pounds) of radiation protection that should be worn by all staff working in this environment (Figure 2A). However, it can increase pressure in the lumbar or cervical discs [8,39]. The impact of anti-X aprons on fitness for work assessment has not been investigated, particularly in subjects with MSDs [19]. The correlation between anti-X apron-wearing and the occurrence of MSDs remains unclear, although the possible discomfort of workers using anti-X aprons appears more evident. Although further studies are needed to clarify the role of these protective devices in the generation of MSDs and to offer specific ergonomic solutions for IRs, it is now recommended to use a two-part coat or one with a suitable belt (Figure 2B). Therefore, the weight is distributed across the shoulders and waist. Careful selection of personal protective garments is thus important and they must fit properly [8]. The shielding material for protective aprons has evolved into lighter weight, composite, or fully lead-free materials while still providing similar protection [40]. Although these materials decrease the pressure on the spine, they do not avoid additional pressure load, which justifies removing the coat between procedures. Some devices have been designed to completely avoid the apron [41]. Freestanding, suspended, or movable shields exist, and can be positioned behind the operator to provide substantial protection. However, attention must be paid to avoid uncomfortable body positions while using such devices because of the reduction in space. Leaded glasses with large lenses and protective side shields are also recommended but adoption is limited due to their weight and discomfort.

**Figure 3:**
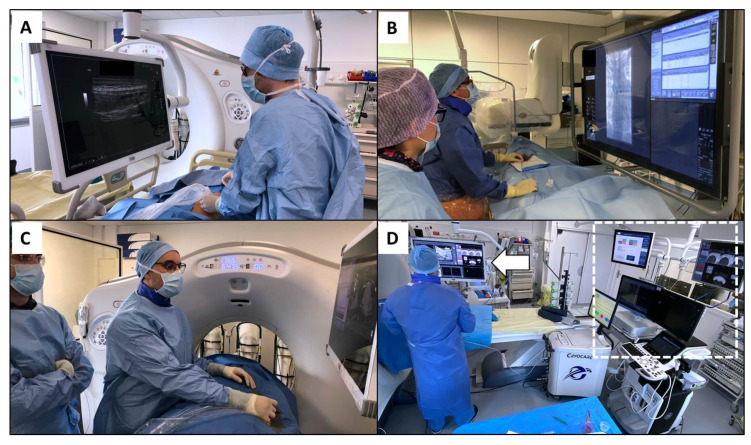
During procedures performed under ultrasound (**A**), X-rays (**B**), or under computed tomography scan (**C**), the screens should be placed in front of the operator to allow a downward viewing angle of 10–15°, which improves the position of the operator’s cervical spine during the whole procedure. Ceiling-mounted monitors can be placed in a wide variety of positions while gathering all the information needed by the operator, including the electromagnetic navigation system, and reducing the congestion of space (**D**, arrow). Head and body movements used to navigate and search among the screens of all the systems required are therefore limited (to the right, as shown by the dashed line).

Special attention should also be paid to the position of the cervical spine, which is often extended and rotated in the angio suite due to the positions of devices and monitors. Ideally, the screen should be in front of the operator to avoid the combined stress of rotation and extension (Figure 3). The monitor should be placed just below eye level [11,32]. The reason for this is that the neck muscles are relaxed at a downward viewing angle of 10°–15°. This position can improve operator comfort by further reducing axial stress during procedures. Ceiling-mounted monitors have the added benefit of being able to be placed in a wide variety of positions [42]. Using a large screen and a broadcast video system may allow an ergonomic multimodal visualization by gathering all information provided by the systems used in the angio suite, such as ultrasound, X-rays, picture archiving and communication system and CT scan (Figure 3D). It helps to limit the risk of postural and visual fatigue by reducing the head and body movements used to navigate and search screens [32,43]. This can further contribute to dose reduction for the patient and staff by allowing digital magnification [44].

Among the items particularly important in the angio suite, the height of the table must be adjusted to allow the elbow joint to remain in a neutral position for most of the operating time to avoid the operator bending forward (Figure 1A). Surgical tables positioned at a height up to 5 cm above the elbow height improves the position of operators (Figure 2B). A study performed during laparoscopic procedures demonstrated that this position allows the biceps brachii to remain at less than 15% of maximum muscle activity while reducing back, shoulder, and wrist discomfort [45]. Moreover, it was demonstrated that standing on a softer surface is more comfortable and less fatiguing than standing on hard floor for prolonged periods of time. Mats or insoles may help to address this issue [46].

### 4.3. Procedures Performed under Computed Tomography and Positron Emission Tomography Scan

Computed tomography (CT) is used to guide a wide range of interventional procedures in various locations ranging from biopsies to ablations [47]. Likewise, positron emission tomography can be used in some indications [48,49]. Apart from the requirements related to radiation exposure, which are the same as in the angio suite, the design of these systems implies that the operator must work near or in the CT tunnel in an awkward or poor posture. As such, the operator must often lean or bend to accomplish procedures (Figure 1B). Interestingly, no ergonomic evaluation has been carried out specifically with these systems. Surgical nippers, advanced visualization, fusion imaging, and electromagnetic navigation can help reduce procedural time while improving IR position (Figure 3D) [50,51].

### 4.4. Magnetic Resonance Imaging Guided Interventions

Performing procedures under magnetic resonance (MR) guidance eliminates the need for protective lead aprons. Compared with CT scan, open field magnets can facilitate the procedure by providing wide access to the bore, improving the position of IRs [52]. However, working under strong magnetic fields has specific requirements [53]. Safety issues include the biological effects of magnetic fields, burns and hearing damage, projectile effects, and compatibility of peripheral equipment. Noise can be a major problem for IRs operating in this environment [54]; It can affect concentration and productivity [32]. Appropriate materials should be selected for operator protection, such as noise reduction headphones, as well as ceiling, floor, and walls to control noise. Regular maintenance of the equipment is also recommended.

## 5. Future Developments

### 5.1. Prevention and Physical Exercise

There is a lack of evidence for scientifically proven methods for the prevention of MSDs, not only in interventional radiology [8]. However, back pain may be mitigated and reduced by applying practical recommendations such as keeping the spine supple and back muscles strong and fatigue-resistant; avoiding spending long periods of time in lordotic or fully flexed positions, as well as rapid and awkward bending movements, especially in the early morning; lifting slowly with the spine balanced and slightly bent, muscles relaxed, and the weigh close to and in front of the body; building up the back’s strength slowly when starting an activity; and sleeping on one’s side rather than on the back. The implementation of such physical exercise training to develop strength, resistance, coordination, and stabilization in interventional radiology might be interesting to prevent MSDs but confounding factors will render its evaluation challenging [55].

### 5.2. Training

The effects of training on the occurrence of musculoskeletal pain and discomfort has been demonstrated but need to be further evaluated in interventional radiology. A better knowledge of ergonomics can considerably reduce MSDs and must be implemented early in the career [56]. To prevent or mitigate back pain, the most basic method of prevention is to identify and stop performing the activity responsible for the pain [8]. The implementation of an ergonomics program in the IR course appears valuable at the earliest phases of medical training. Changing posture, reduction in the time in the interventional room, and a reduction in the overall caseload may reduce the occurrence of MSDs [7]. When possible, sitting is better than standing from an ergonomic perspective [9]. Micro breaks of two minutes every 20–40 min may be considered [57].

### 5.3. Specialization and Design of Interventional Equipments

In interventional radiology, the integration of ergonomics in the workplace must balance the ergonomic disadvantages of shielding [11]. Specific recommendations for the angiographic room must now be provided to preserve the well-being of IRs [58]. Firstly, equipment dedicated to IRs is needed [59]. It was demonstrated that specialization of the systems by assigning image-guided interventions to the interventional suite and diagnostic imaging to the dedicated equipment improves efficiency by maximizing use while limiting spatial issues of walking between different physical workspaces to perform interventions [60]. Hybrid CT/angiography system (angio-CT) is a promising option integrating all imaging modalities within the same room [60]. All are interfaced on the same monitor. This may help to reduce the movement of staff and patients by improving workflow and production. Radiation experts, physicians, and administration must further work together to design the next generation of ergonomic and specialized interventional suites [10].

### 5.4. Robotics and New Technologies

The development of robotics can improve the accuracy of procedures while limiting radiation exposure to staff [61]. This may open up new horizons for IRs in terms of ergonomics such as those already observed in surgery [62]. The new generation of robotic cone-beam computed tomography system, known as the hybrid operating room, helps limit radiation exposure and improve user experience and workflow efficiency, even in complex working positions at the head, neck, or left side of the patient, while increasing the adoption of advanced image guidance in daily practice [63]. Exoskeletons may help to reduce loads by bearing the weight of the shielding but they have to be further evaluated as working tasks in interventional radiology generally involve multiple movements, which may be restricted by such a system [64]. Additionally, augmented or mixed reality may enhance guidance by allowing head-up display or advanced 3D visualization [65,66].

## 6. Conclusions

The prevalence and impact of MSDs on IR practice requires increased awareness and prevention. Improvements in the ergonomics of interventional radiology have the potential to alleviate these symptoms, improve productivity and performance, reduce time off work, extend careers, and ultimately improve patient care. Training programs focused on ergonomics are now mandatory as is an evolution in the design of medical devices and interventional suites.

## Data Availability

Not applicable.

## References

[1] Filippiadis D., Tutton S., Kelekis A. (2017). Pain management: The rising role of interventional oncology. Diagn. Interv. Imaging.

[2] Cornelis F. (2017). The interventional oncologist: The fourth musketeer of cancer care. Diagn. Interv. Imaging.

[3] Vroomen L., Petre E., Cornelis F., Solomon S., Srimathveeravalli G. (2017). Irreversible electroporation and thermal ablation of tumors in the liver, lung, kidney and bone: What are the differences?. Diagn. Interv. Imaging.

[4] Cornelis F.H., Joly Q., Nouri-Neuville M., Ben-Ammar M., Kastler B., Kastler A., Amoretti N., Hauger O. (2019). Innovative Spine Implants for Improved Augmentation and Stability in Neoplastic Vertebral Compression Fracture. Medicina.

[5] Cornelis F.H., Tutton S., Filippiadis D., Kelekis A. (2017). Metastatic Osseous Pain Control: Bone Ablation and Cementoplasty. Semin. Interv. Radiol..

[6] Klein L.W., Miller D.L., Balter S., Laskey W., Haines D., Norbash A., Mauro M.A., Goldstein J.A. (2009). Joint Inter-Society Task Force on Occupational Hazards in the Interventional Laboratory. Occupational Health Hazards in the Interventional Laboratory: Time for a Safer Environment. J. Vasc. Interv. Radiol..

[7] Dalager T., Søgaard K., Boyle E., Jensen P.T., Mogensen O. (2019). Surgery Is Physically Demanding and Associated with Multisite Musculoskeletal Pain: A Cross-Sectional Study. J. Surg. Res..

[8] Dixon R.G., Khiatani V., Statler J.D., Walser E.M., Midia M., Miller D.L., Bartal G., Collins J.D., Gross K.A., Stecker M.S. (2017). Society of Interventional Radiology: Occupational Back and Neck Pain and the Interventional Radiologist. J. Vasc. Interv. Radiol..

[9] Aaron K.A., Vaughan J., Gupta R., Ali N.-E.-S., Beth A.H., Moore J.M., Ma Y., Ahmad I., Jackler R.K., Vaisbuch Y. (2021). The risk of ergonomic injury across surgical specialties. PLoS ONE.

[10] Benjamin J.L., Meisinger Q.C. (2018). Ergonomics in the Development and Prevention of Musculoskeletal Injury in Interventional Radiologists. Tech. Vasc. Interv. Radiol..

[11] Knuttinen M.-G., Zurcher K.S., Wallace A., Doe C., Naidu S.G., Money S.R., Rochon P.J. (2021). Ergonomics in IR. J. Vasc. Interv. Radiol..

[12] Baethge C., Goldbeck-Wood S., Mertens S. (2019). SANRA—a scale for the quality assessment of narrative review articles. Res. Integr. Peer Rev..

[13] Machan L. (2002). Risks of Back Injury. J. Vasc. Interv. Radiol..

[14] Epstein S., Sparer E.H., Tran B.N., Ruan Q.Z., Dennerlein J.T., Singhal D., Lee B.T. (2018). Prevalence of Work-Related Musculoskeletal Disorders Among Surgeons and Interventionalists: A Systematic Review and Meta-analysis. JAMA Surg..

[15] Wells A.C., Kjellman M., Harper S.J.F., Forsman M., Hallbeck M.S. (2019). Operating hurts: A study of EAES surgeons. Surg. Endosc..

[16] McDonald M.E., Ramirez P.T., Munsell M.F., Greer M., Burke W.M., Naumann W.T., Frumovitz M. (2014). Physician pain and discomfort during minimally invasive gynecologic cancer surgery. Gynecol. Oncol..

[17] Adams S.R., Hacker M.R., McKinney J.L., Elkadry E.A., Rosenblatt P.L. (2013). Musculoskeletal Pain in Gynecologic Surgeons. J. Minim. Invasive Gynecol..

[18] Alleblas C.C.J., de Man A.M., Haak L.V.D., Vierhout M.E., Jansen F.W., Nieboer T.E. (2017). Prevalence of Musculoskeletal Disorders Among Surgeons Performing Minimally Invasive Surgery. Ann. Surg..

[19] Monaco M.G.L., Carta A., Tamhid T., Porru S. (2020). Anti-X Apron Wearing and Musculoskeletal Problems Among Healthcare Workers: A Systematic Scoping Review. Int. J. Environ. Res. Public Health.

[20] Moore B., Vansonnenberg E., Casola G., Novelline R.A. (1992). The relationship between back pain and lead apron use in radiologists. Am. J. Roentgenol..

[21] Goldstein J.A., Balter S., Cowley M., Hodgson J., Klein L.W. (2004). Occupational hazards of interventional cardiologists: Prevalence of orthopedic health problems in contemporary practice. Catheter. Cardiovasc. Interv..

[22] Orme N.M., Rihal C.S., Gulati R., Holmes D.R., Lennon R.J., Lewis B.R., McPhail I.R., Thielen K.R., Pislaru S.V., Sandhu G.S. (2015). Occupational Health Hazards of Working in the Interventional Laboratory. J. Am. Coll. Cardiol..

[23] Sutton E., Irvin M., Zeigler C., Lee G., Park A. (2014). The ergonomics of women in surgery. Surg. Endosc..

[24] Hemal A., Srinivas M., Charles A. (2001). Ergonomic Problems Associated with Laparoscopy. J. Endourol..

[25] Wong K., Grundfast K.M., Levi J.R. (2017). Assessing work-related musculoskeletal symptoms among otolaryngology residents. Am. J. Otolaryngol..

[26] Davis W.T., Fletcher S.A., Guillamondegui O.D. (2014). Musculoskeletal occupational injury among surgeons: Effects for patients, providers, and institutions. J. Surg. Res..

[27] Sergesketter A.R., Lubkin D.T., Shammas R.L., Krucoff K.B., Peskoe S.B., Risoli T., Endres K., Hollenbeck S.T. (2019). The Impact of Ergonomics on Recruitment to Surgical Fields: A Multi-Institutional Survey Study. J. Surg. Res..

[28] Rotenstein L.S., Torre M., Ramos M.A., Rosales R.C., Guille C., Sen S., Mata D.A. (2018). Prevalence of Burnout Among Physicians: A Systematic Review. JAMA.

[29] De Hert S. (2020). Burnout in Healthcare Workers: Prevalence, Impact and Preventative Strategies. Local Reg. Anesth..

[30] Davila V.J., Meltzer A.J., Hallbeck M.S., Stone W.M., Money S.R. (2019). Physical discomfort, professional satisfaction, and burnout in vascular surgeons. J. Vasc. Surg..

[31] Stucky C.-C.H., Cromwell K.D., Voss R.K., Chiang Y.-J., Woodman K., Lee J.E., Cormier J.N. (2018). Surgeon symptoms, strain, and selections: Systematic review and meta-analysis of surgical ergonomics. Ann. Med. Surg..

[32] Goyal N., Jain N., Rachapalli V. (2009). Ergonomics in radiology. Clin. Radiol..

[33] Smith A.C., Wolf J.G., Xie G.-Y., Smith M.D. (1997). Musculoskeletal pain in cardiac ultrasonographers: Results of a random survey. J. Am. Soc. Echocardiogr..

[34] Barros-Gomes S., Orme N., Nhola L.F., Scott C., Helfinstine K., Pislaru S.V., Kane G.C., Singh M., Pellikka P.A. (2019). Characteristics and Consequences of Work-Related Musculoskeletal Pain among Cardiac Sonographers Compared with Peer Employees: A Multisite Cross-Sectional Study. J. Am. Soc. Echocardiogr..

[35] Magnavita N., Bevilacqua L., Mirk P., Fileni A., Castellino N. (1999). Work-Related Musculoskeletal Complaints in Sonologists. J. Occup. Environ. Med..

[36] Christenssen W.D. (2001). Stretch Exercises. J. Diagn. Med. Sonogr..

[37] Mirowski M.M. (2020). New-old methods of reducing and monitoring X-ray exposure in the interventional radiology environment. Med. Pract..

[38] Tetteh E., Sarker P., Radley C., Hallbeck M.S., Mirka G.A. (2020). Effect of surgical radiation personal protective equipment on EMG-based measures of back and shoulder muscle fatigue: A laboratory study of novices. Appl. Ergon..

[39] Ross A.M., Segal J., Borenstein D., Jenkins E., Cho S. (1997). Prevalence of Spinal Disc Disease Among Interventional Cardiologists. Am. J. Cardiol..

[40] Miller D.L., Vañó E., Bartal G., Balter S., Dixon R., Padovani R., Schueler B., Cardella J.F., De Baère T. (2009). Occupational Radiation Protection in Interventional Radiology: A Joint Guideline of the Cardiovascular and Interventional Radiology Society of Europe and the Society of Interventional Radiology. Cardiovasc. Interv. Radiol..

[41] Marichal D.A., Anwar T., Kirsch D., Clements J., Carlson L., Savage C., Rees C.R. (2011). Comparison of a Suspended Radiation Protection System versus Standard Lead Apron for Radiation Exposure of a Simulated Interventionalist. J. Vasc. Interv. Radiol..

[42] Sikkink C., Reijnen M., Zeebregts C. (2008). The Creation of the Optimal Dedicated Endovascular Suite. Eur. J. Vasc. Endovasc. Surg..

[43] Harisinghani M.G., Blake M.A., Saksena M., Hahn P.F., Gervais D., Zalis M., Fernandes L.D.S.D., Mueller P.R. (2004). Importance and Effects of Altered Workplace Ergonomics in Modern Radiology Suites1. Radiography.

[44] Barral M., Gardavaud F., Lassalle L., Ben Ammar M., Najdawi M., Razakamanantsoa L., Renard-Penna R., Cussenot O., Cornelis F.H. (2021). Limiting radiation exposure during prostatic arteries embolization: Influence of patient characteristics, anatomical conditions, and technical factors. Eur. Radiol..

[45] Van Veelen M., Kazemier G., Koopman J., Goossens R., Meijer D. (2002). Assessment of the Ergonomically Optimal Operating Surface Height for Laparoscopic Surgery. J. Laparoendosc. Adv. Surg. Tech..

[46] King P.M. (2002). A comparison of the effects of floor mats and shoe in-soles on standing fatigue. Appl. Ergon..

[47] Leng S., Christner J.A., Carlson S.K., Jacobsen M., Vrieze T.J., Atwell T.D., McCollough C.H. (2011). Radiation Dose Levels for Interventional CT Procedures. Am. J. Roentgenol..

[48] Solomon S.B., Cornelis F.H. (2016). Interventional Molecular Imaging. J. Nucl. Med..

[49] Bogoni M., Cerci J.J., Cornelis F.H., Nanni C., Tabacchi E., Schöder H., Shyn P.B., Sofocleous C.T., Solomon S.B., Kirov A.S. (2021). Practice and prospects for PET/CT guided interventions. Q. J. Nucl. Med. Mol. Imaging.

[50] Meyer B.C., Peter O., Nagel M., Hoheisel M., Frericks B.B., Wolf K.-J., Wacker F.K. (2008). Electromagnetic field-based navigation for percutaneous punctures on C-arm CT: Experimental evaluation and clinical application. Eur. Radiol..

[51] Abi-Jaoudeh N., Kruecker J., Kadoury S., Kobeiter H., Venkatesan A.M., Levy E., Wood B.J. (2012). Multimodality Image Fusion–Guided Procedures: Technique, Accuracy, and Applications. Cardiovasc. Interv. Radiol..

[52] Ahrar K., Ahrar J.U., Javadi S., Pan L., Milton D.R., Wood C.G., Matin S.F., Stafford R.J. (2013). Real-Time Magnetic Resonance Imaging–Guided Cryoablation of Small Renal Tumors at 1.5 T. Investig. Radiol..

[53] Pickup L., Nugent B., Bowie P. (2019). A preliminary ergonomic analysis of the MRI work system environment: Implications and recommendations for safety and design. Radiography.

[54] Bock M., Wacker F.K. (2008). MR-guided intravascular interventions: Techniques and applications. J. Magn. Reson. Imaging.

[55] Sjøgaard G., Christensen J.R., Justesen J.B., Murray M., Dalager T., Fredslund G.H., Søgaard K. (2016). Exercise is more than medicine: The working age population’s well-being and productivity. J. Sport Health Sci..

[56] Robertson M.M., O’Neill M.J. (2003). Reducing Musculoskeletal Discomfort: Effects of an Office Ergonomics Workplace and Training Intervention. Int. J. Occup. Saf. Ergon..

[57] Hallbeck M., Lowndes B., Bingener J., Abdelrahman A., Yu D., Bartley A., Park A. (2017). The impact of intraoperative microbreaks with exercises on surgeons: A multi-center cohort study. Appl. Ergon..

[58] Cornelis F.H., Najdawi M., Ben Ammar M., Nouri-Neuville M., Lombart B., Lotz J.-P., Cadranel J., Barral M. (2020). Integrative Medicine in Interventional Oncology: A Virtuous Alliance. Medicina.

[59] Cornelis F.-H., Solomon S.-B. (2020). Image guidance in interventional radiology: Back to the future?. Diagn. Interv. Imaging.

[60] Feinberg N., Funaki B., Hieromnimon M., Guajardo S., Navuluri R., Zangan S., Lorenz J., Ahmed O. (2020). Improved Utilization Following Conversion of a Fluoroscopy Suite to Hybrid CT/Angiography System. J. Vasc. Interv. Radiol..

[61] Cornelis F.H., Takaki H., Laskhmanan M., Durack J.C., Erinjeri J.P., Getrajdman G.I., Maybody M., Sofocleous C.T., Solomon S.B., Srimathveeravalli G. (2014). Comparison of CT Fluoroscopy-Guided Manual and CT-Guided Robotic Positioning System for In Vivo Needle Placements in Swine Liver. Cardiovasc. Interv. Radiol..

[62] Franasiak J., Craven R., Mosaly P., Gehrig P.A. (2014). Feasibility and Acceptance of a Robotic Surgery Ergonomic Training Program. JSLS J. Soc. Laparoendosc. Surg..

[63] Santos J.S., Uusi-Simola J., Kaasalainen T., Aho P., Venermo M. (2020). Radiation Doses to Staff in a Hybrid Operating Room: An Anthropomorphic Phantom Study with Active Electronic Dosimeters. Eur. J. Vasc. Endovasc. Surg..

[64] De Vries A.W., Krause F., De Looze M.P. (2021). The effectivity of a passive arm support exoskeleton in reducing muscle activation and perceived exertion during plastering activities. Ergonomics.

[65] Park B.J., Hunt S.J., Martin C., Nadolski G.J., Wood B.J., Gade T.P. (2020). Augmented and Mixed Reality: Technologies for Enhancing the Future of IR. J. Vasc. Interv. Radiol..

[66] Bin Helayel H., Al-Mazidi S., Al Akeely A. (2021). Can the Three-Dimensional Heads-Up Display Improve Ergonomics, Surgical Performance, and Ophthalmology Training Compared to Conventional Microscopy?. Clin. Ophthalmol..

